# Severe, Treatment-Refractory Periodontitis and Vitamin D Deficiency: A Multidisciplinary Case Report

**DOI:** 10.1155/2022/6469214

**Published:** 2022-10-11

**Authors:** Figliuzzi Michele Mario, Parentela Luca, Aiello Domenico, Altilia Simone

**Affiliations:** Department of Health Sciences, “Magna Graecia” University, Catanzaro 88100, Italy

## Abstract

**Introduction:**

Vitamin D deficiency and periodontitis are common diseases among people. These conditions interact with each other and worsen the patient's health. Modern dentistry aims to rehabilitate oral health and bring it back to the original state or even improve aesthetics. Without analysing the general health conditions of patients and without a multidisciplinary approach, it is sometimes not possible to solve a case such as we describe. This study shows how a vitamin D deficit can influence the choice of dental treatment. The patient, a 40-year-old woman, in generally good health, came to our observation complaining about pain during mastication, and profuse bleeding during home hygiene maneuvers. She wished to solve this situation.

**Materials and Methods:**

Since the patient did not respond well to the initial periodontal therapy, we carried out some accurate research concerning the patient's previous clinical history, and as we suspected that a vitamin D deficit might be involved, a chemical test for vitamin D was carried out and the results confirmed our suspicions. The team programmed the following therapeutical plan: Phase 1—rehabilitation of normal values of vitamin D with the support of an endocrinologist; Phase 2—full mouth disinfection; Phase 3—periodontal surgical restorative therapy; and Phase 4—orthodontic therapy and fixed prosthetics rehabilitation.

**Results:**

The results for this clinical case were extremely satisfying; we were able to bring the periodontal illness under control; resolve the orthodontic problems; and rehabilitate the patient both functionally and aesthetically.

**Conclusion:**

Thanks to the collaboration and communication between specialists from different sectors of medicine and dentistry, the clinical case was solved with absolutely satisfactory results.

## 1. Introduction

Vitamin D deficit is very common in the population and it is becoming more predominant in the groups of older and elderly people [1–3].

A vitamin D deficit leads to inadequate absorption of calcium and phosphate. This consequently gives rise to a reduction in the levels of calcium in the blood plasma, thus stimulating the parathyroid hormone, which acts to realign the calcium levels in the blood at the expense of the bones.

Periodontitis is a very common disease affecting over 50% of adults and a significant percentage of this group suffers from a very severe form [4, 5].

Periodontitis is a multifactorial disease primarily caused by plaque microorganisms, modified by the immune inflammatory response to chronic infection, which leads to the destruction of periodontal tissues in a susceptible host.

It is very well known that vitamin D plays a vital role in bone homeostasis and immunity. There is a biological rationale to suspect that vitamin D deficiency could negatively affect the periodontium [6–8].

When considering the periodontal area, normal levels of vitamin D reduce the expression of interleukin-8 and interleukin-6, reducing the virulence of *Porphyromonas gingivalis* [9].

Other natural substances, such as probiotics, could play a similar role since these seem to contribute to the maintenance of tissue homeostasis [10–13].

The two conditions, which are epidemiologically relevant, seem to interact empirically; vitamin D deficits are correlated to a higher prevalence of periodontitis and periodontopathic patients show lower levels of this vitamin [14, 15].

Vitamin D could play a role in the treatment of periodontitis [16–18].

Moreover, it seems that polymorphisms of vitamin D receptors are involved in the etiopathogenesis of periodontitis, but this point is controversial [19–21].

Today's dentistry is not only carried out to restore the functionality of the stomatognathic apparatus but also aims to improve the general health of the patient and to restore and/or improve the aesthetics of the patient; therefore, it is advisable that clinicians become familiar with all the disciplines that make up dentistry to guarantee an adequate performance [22].

The objective of this study is to demonstrate how the patient's general health influences oral health, and especially the periodontium, and that an improvement in general health can sometimes be an effective way forward to achieving a satisfactory resolution, using multidisciplinary treatment, and thus obtaining an aesthetical and functional rehabilitation of the patient.

## 2. Materials and Methods

### 2.1. The Clinical Case

When the patient came to our notice, they presented a severe impairment of the entire stomatognathic apparatus with hyperaemic, oedemic gums, and severe functional and aesthetic problems, severely impaired dental arches, serious malocclusion, and diastema [12, 13].

**Figure 1:**
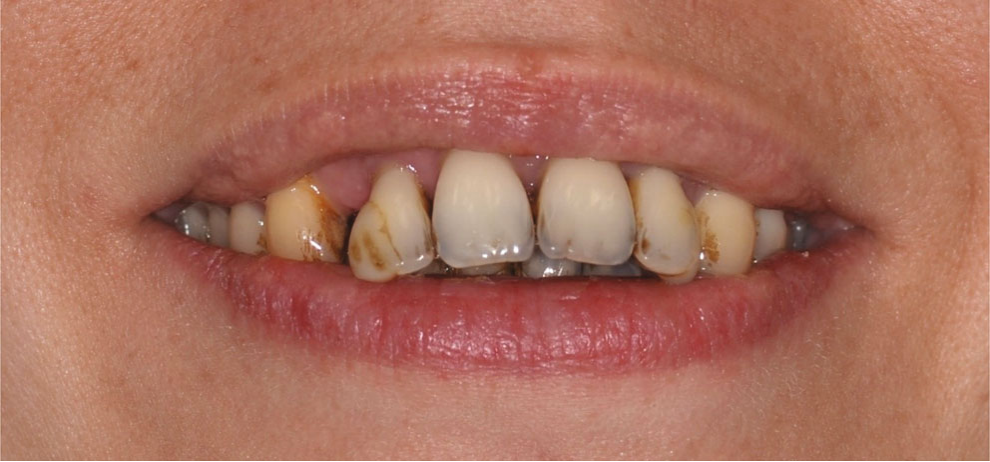
Clinical case: frontal view.

**Figure 2:**
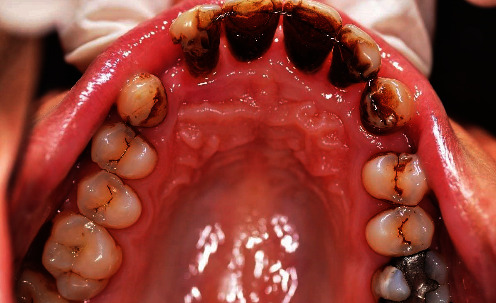
Clinical case: occlusal view, lower jaw.

**Figure 3:**
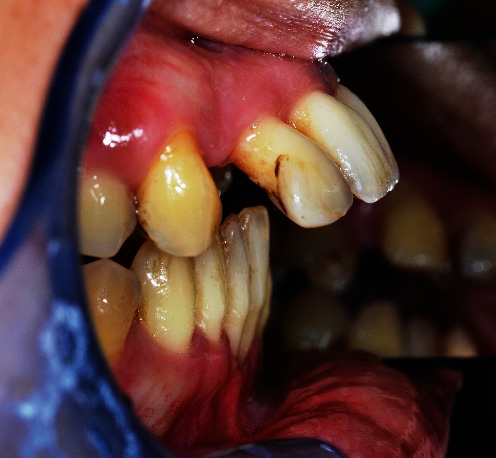
Clinical case: lateral view.

The patient presented with an increased overjet due to a significant proclination of the upper incisors and a deviation of the upper midline to the left. This situation was probably due to a horizontal bone reabsorption associated with labial interposition (Figures 1–3).

The patient told us that they had undergone a number of periodontal therapies (scaling and root planing and non-surgical periodontal therapy) in the past but that they had never obtained satisfactory results.

**Figure 4:**
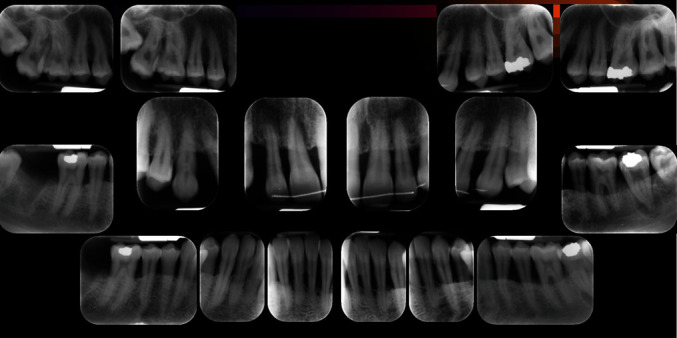
Rx full endoral clinical case: Rx.

After carrying out the anamnesis, the patient underwent a clinical check-up, X-rays (Figure 4), and a periodontal scan.

**Figure 5:**
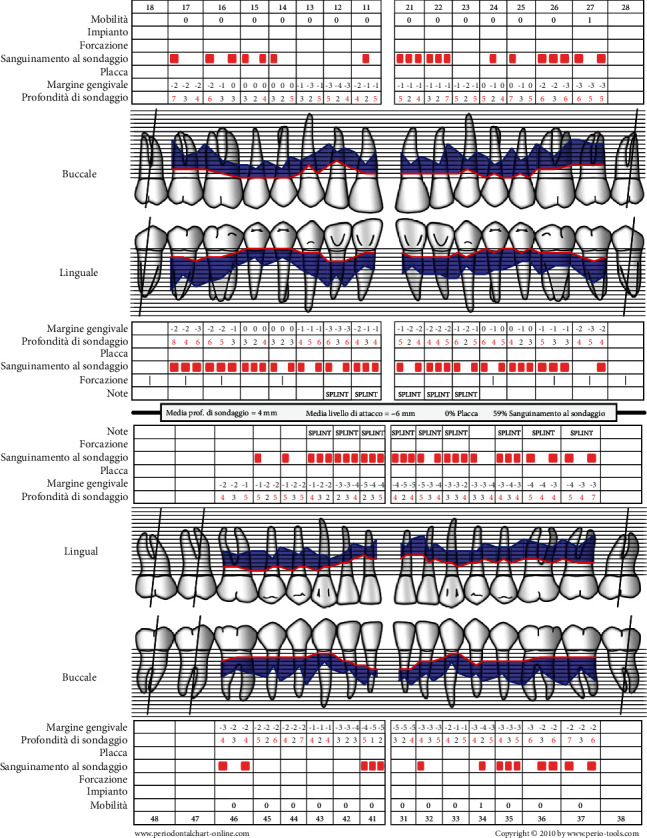
Probing with periodontal indices.

The patient's periodontal values (Figure 5) were followed and measured before the rehabilitation therapy (and again at 6 and 12 months after completion of the therapy):

- Plaque index presence/absence of visible plaque in 6 points of each tooth [23].
- Bleeding on probing (BoP) presence/absence of bleeding when using probes in 6 points for each tooth [24].
- Full mouth plaque score (FMPS) expressed as a percentage [25].
- Full mouth bleeding score (FMBS) for BoP expressed as a percentage.
- Probing depth registered in millimetres in 6 points for each tooth [26, 27].

**Figure 6:**
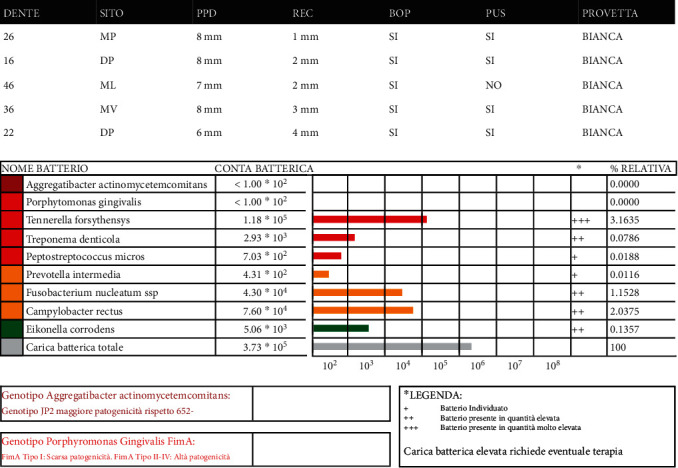
Microbiological test results.

**Figure 7:**
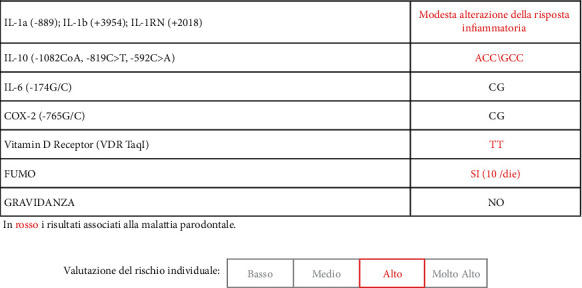
Genetic test results.

Both microbiological tests (biomolecular diagnostics) and genetic tests (genetical periodontic screening) were carried out (Figures 6 and 7).

Hematochemical tests were prescribed and, in particular, the one to test vitamin D levels.

The exam of the levels of vitamin D in the plasma were outside the normal levels.

**Figure 8:**
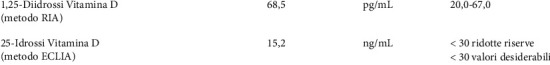
Laboratory test results related to vitamin D: vitamin D values.

The patient suffered from a severe vitamin D absorption deficit (Figure 8).

The patient underwent a bone densitometry exam on the femur.

Computerised bone mineralometry confirmed the low bone mineralisation levels.

### 2.2. The Therapeutical Plan

From the results of the clinical visit, the X-rays, the microbiological tests, the genetic test which showed an alteration of interleukin L1, and the laboratory tests, the following therapeutic plan (based on scientific evidence) was drawn up [28–32].

- Phase 1: rehabilitation of normal values of vitamin D with the support of an endocrinologist; A vitamin D supplementation protocol made it possible to re-establish the normal systemic homeostasis of the organism:
- 1,25-OH vitamin D: 45.1 pg/ml.
- 25-OH-vitamin D: 26.3 pg/ml.
- Phase 2: full mouth disinfection.
- Phase 3: periodontal surgical restorative therapy.
- Phase 4: orthodontic therapy and fixed prosthetics rehabilitation.

The dental treatment methods are described in detail as follows:

- Instruction and motivation.
- Causal periodontal therapy associated with pharmacological antibiotic and antiseptic therapy using chlorhexidine 0.2%.

Full mouth disinfection with pharmacological antibiotic therapy using tetracillin chloridate 250 mg 3 times a day every 8 hours (Ambramicina Scharper SpA, Milan, Italy) and amoxicillin with clavulanic acid (Augmentin, Glaxosmithkline SpA, Verona, Italy) 1 g per day for 8 days. Chlorexidine 0.2% mouthwash (Cliadent 0.2% Budetta Farma s.r.l., Salerno, Italy) 2 rinses for 1 minute each per day for 10 days.

The periodontal parameters were normal throughout the period of the study (6 months), the FMPS and the FMBS were less than 20% before the prosthetic therapy and at 6 months from the definitive cementation (Figure 4).

- Splinting from 33 to 43 associated with stripping.
- Devitalisation of 37.
- Splinting of 35, 36, and 37.
- Extraction of 28 and 38.
- Grafting with biomaterial followed by an implant on 47.
- Fixed orthodontic therapy.

**Figure 9:**
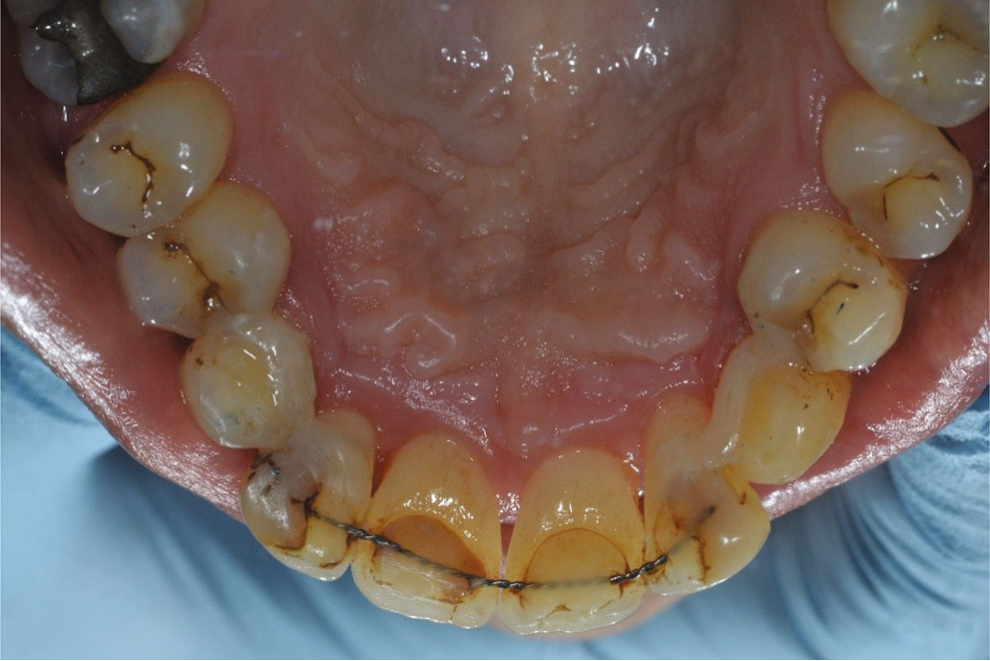
Clinical case: occlusal view, lower jaw, orthodontic therapy.

**Figure 10:**
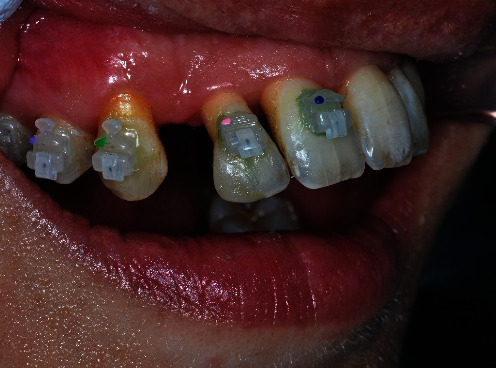
Clinical case: 3/4 view, orthodontic therapy.

When the tissues were stabilised, the orthodontic therapy was initialised using a fixed multibracket and aesthetic orthodontic therapy according to the prescription of Prof. W. Roth. Over the timespan of six months the orthodontic treatment was carried out with a sequence of standard arches (0.014 niti, 0.016 niti, 0.017 × 0.025 niti, 0.019 × 0.025 niti, e 0.019 × 0.025 ss plus an elastic chain) with the aim of retroclininig the upper incisors and resolving the position of the midline (Figures 9 and 10).

**Figure 11:**
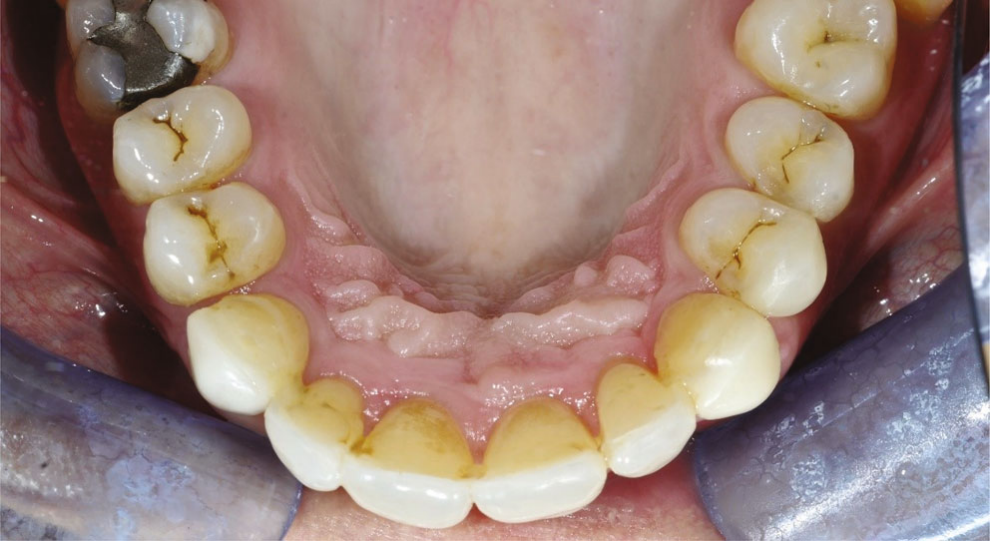
Clinical case: occlusal view, lower jaw, prosthetic therapy.

- Functional prosthetic and aesthetic rehabilitation
- After completing the orthodontic therapy (Figure 11) prosthetic rehabilitation was carried out.

## 3. Results

**Figure 12:**
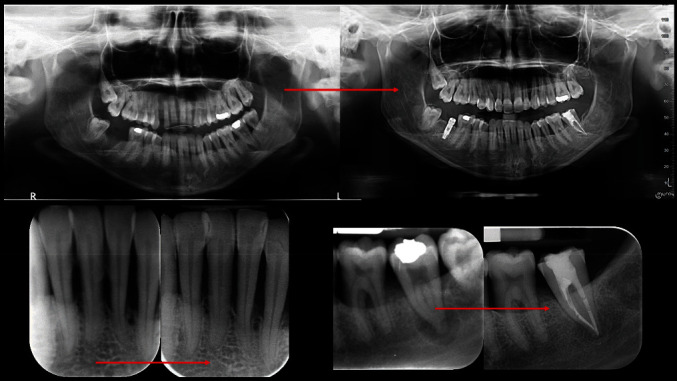
Clinical case: Rx pre- and post-treatment.

**Figure 13:**
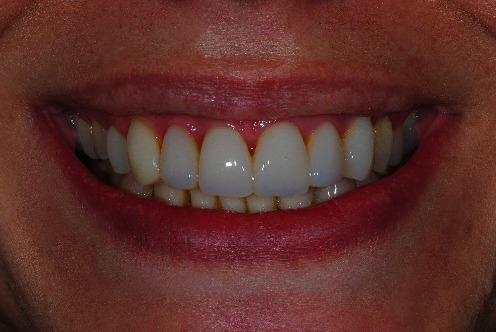
Clinical case: frontal view, at the end of the treatment.

**Figure 14:**
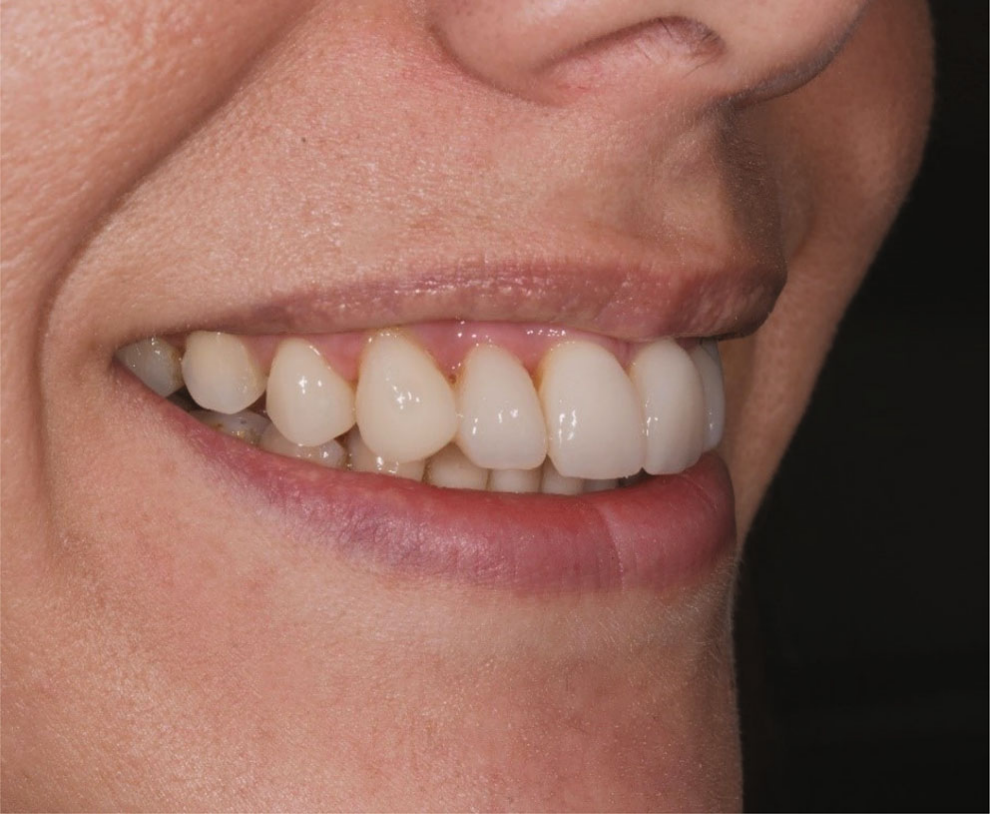
Clinical case: 3/4 view, at the end of the treatment.

We were able to:

- Bring the periodontal illness under control.
- Take the FMBS and the FMPS down to zero.
- Resolve the orthodontic problems.
- Rehabilitate the patient both functionally and aesthetically (Figures 12–14).

## 4. Discussion

This work aims at explaining how a well-considered and multidisciplinary approach, based on a rigorous scientific method, which considers the organism's high reparative potential, can resolve a highly compromised situation in a patient with severe periodontitis associated with a vitamin D deficit, malocclusions, and diastemas. In today's interdisciplinary world, treatment planning must start with well-defined aesthetic goals.

By starting with aesthetics and considering the impact on function, structure, and biology, the clinician will be able to use the various disciplines of dentistry to deliver the highest level of dental care to each patient.

Several dentists worked on the same case, each acting in their own specific field of action but with constant communication and synergy of intent.

Furthermore, this study highlights how important it is to consider other health parameters as the case was solved thanks to the intuition of the possibility of a vitamin D deficit with the relative confirmation from laboratory analyses.

Few cases of this type are reported in literature and therefore this study will serve as a guide to solving similarly complex cases. Correlation of vitamin D with periodontitis is an interesting topic, according to literature [7, 15], which requires more study to be clarified. This study, in particular, is yet another piece of evidence showing that, in our opinion, adequate serum levels of vitamin D could be essential in some cases for the control of periodontal disease.

The application of postbiotic gels to activate a proactive phase in combination with vitamin D treatment could be useful [33].

## 5. Conclusion

Once again, the importance of knowing and evaluating the patient's general state of health must be emphasized in order to be able to identify any systemic causes of a periodontal problem; in this case, it was important to identify the vitamin D deficiency, a widely spread condition among the population.

We believe that, today, a multidisciplinary approach to solving such complicated clinical cases is essential. Even if the literature is not exhaustive on the topic, we believe that vitamin D deficiency should be suspected, investigated, and eventually treated, in the case of periodontal situations that do not improve after conventional treatment. We sincerely hope that this topic will be further researched in the near future, producing new studies that could consider, for example, the use of other support molecules, such as postbiotics.

## Data Availability

Data supporting this research article are available from the corresponding author or first author on reasonable request.
